# Sexually transmitted infections and the HPV-related burden: evolution of Italian epidemiology and policy

**DOI:** 10.3389/fpubh.2024.1336250

**Published:** 2024-03-15

**Authors:** Silvia Gazzetta, Francesca Valent, Alessia Sala, Lorenza Driul, Laura Brunelli

**Affiliations:** ^1^Departement of Medicine, University of Udine, Udine, Italy; ^2^Institute of Hygiene and Clinical Epidemiology, Friuli Centrale University Healthcare Trust, Udine, Italy; ^3^Department of Obstetrics and Gynaecology, ASUFC, Ospedale Santa Maria Della Misericordia, Udine, Italy; ^4^Accreditation, Quality and Clinical Risk Unit, Friuli Centrale University Healthcare Trust, Udine, Italy

**Keywords:** sexually transmitted infections, HPV, cervical cancer, screening, vaccination, health policies, sexual and reproductive health, sex education

## Abstract

Sexually transmitted infections (STIs) are a major public health problem worldwide, with a high prevalence between the ages of 15 and 25 in most Western countries. High notification rates of chlamydia, gonorrhea, and syphilis are reported in the WHO European Region, with differences between countries. In Italy, the total number of STIs alerts increased by 18% from 2020 to 2021. HPV is the most common sexually transmitted infection; globally one in seven women is infected by this virus, and certain sexual behaviors are important risk factors for HPV-related cancers, particularly cervical cancer (CC), anogenital cancers and cancers of the head and neck. The burden of CC is relevant worldwide, in particular in Europe CC is the third leading cause of cancer-related deaths in women aged 15–44. This HPV-related tumor is preventable through a combined strategy of vaccination and screening for precursor lesions. In Italy, the coverage of organized screening varies from region to region and the average HPV vaccination rate is still far from the expected optimal threshold of 95% at the age of 12. To address the challenges of health promotion and HPV prevention, priority actions are needed such as: promoting education and information at every level, from schools to healthcare professionals. In Italy, education of adolescents on sexual and reproductive health, still remains critical, regionally inhomogeneous and much lower than in other European countries. Equitable measures need to be taken, and schools are an important place for health promotion activities.

## 1 Introduction

Sexually transmitted infections (STIs) are a major public health problem in both resource-rich and-poor countries (1). Worldwide, more than one million curable STIs are acquired every day, mainly caused by four pathogens: *Chlamydia trachomatis*, *Neisseria gonorrhoeae*, *Treponema pallidum*, and *Trichomonas vaginalis*, with a global incidence of 374 million new cases of these STIs per year. In terms of sexually transmitted viral pathogens, it is estimated that one in seven women is infected with the human papillomavirus (HPV), and more than 500 people have genital herpes virus (HSV) infection. Behavioral, social, and structural determinants of these STIs include human immunodeficiency virus (HIV), which is closely associated with other sexually transmitted infections: STIs are associated with an increased risk of HIV transmission or acquisition, and HIV enhances HPV-induced carcinogenesis (2). Evidence suggests that the highest prevalence of STIs occurs in the first decade after first sexual intercourse, typically between the ages of 15 and 25 years in most Western countries. Most STIs are asymptomatic but can lead to various complications and the burden of morbidity and mortality due to STIs affects quality of life, sexual and reproductive health, and neonatal and child health (1, 3). Worldwide, according to the latest 2020 data, incidence cases of chlamydia in individuals (per 1,000) are 36.0 in women and 29.0 in men; incidence cases of gonorrhea are 19.0 in women and 23.0 in men; incidence rate of active syphilis is 1.8, with no sex difference (4). The epidemiological situation in the WHO European Region, according to a recent review that included data from 49 countries, is as follows: in 2019 Northern European (EU) countries had higher reporting rates for chlamydia, gonorrhea and syphilis than other EU and non-EU countries. For chlamydia, most northern EU countries reported chlamydia notification rates of more than 300 per 100,000 inhabitants. The Western, Southern and Eastern EU countries reported rates for chlamydia of up to 143.2 per 100,000; the reporting rate in Italy was 1.85/100,000. The total number of reported gonorrhea in the EU countries was 31.3/100,000; the notification rate in Italy was 1.36/100,000. For syphilis, higher rates were observed in the Netherlands (104.5), Malta (19.2), while the notification rate in Italy was 3.05 per 100,000 (5). In Italy, the Sentinel Surveillance System reports that the total number of STI alerts increased by 18% in 2021 compared to 2020: chlamydia, gonorrhea and syphilis recorded a significant increase in 2021 compared with 2020, with a greater increase in cases observed in men who have sex with other men. In addition, the prevalence of chlamydia infections in young people aged 15 to 24 is three times higher than in older adults (6). In 2022, the WHO European Region reported an overall crude rate of 12.4 HIV diagnoses per 100,000 population, a slight increase from the 2021 rate (11.9/100,000) (7). In Italy, HIV prevalence among people with a confirmed STI was 14.7% in 2021, about seventy times higher than the estimated prevalence in the general adult population (6). Anogenital warts represent another significant public health problem, but they are not epidemiologically monitored in the EU. Estimates from the United States, Canada, Australia and several high-income European countries suggest an annual incidence of 0.1–0.2%, peaking in teenagers and young adults (8, 9). A study conducted in Italy in 2017 found an incidence of external genital warts of 3.0 cases per 1,000 women in the female population aged 15–64 years (data from 2009 to 2010) (10). There was an upward trend in HPV-related anogenital warts from 2004 to 2018, but a slight decrease has been observed since then, probably due to the HPV vaccination campaigns in both women and men (6). The interpretation of epidemiological data on STIs must consider the great heterogeneity in terms of access to testing, testing procedures, diagnostic techniques, surveillance systems and reporting practices, which can distort comparisons between countries (5).

## 2 The silent HPV burden

The most common sexually transmitted infection worldwide is caused by HPV, which has a very negative impact on people’s social lives. More than 80% of sexually active people are infected with HPV at least once in their lives, even if they do not necessarily develop the associated disease (3, 11). According to the International Human Papillomavirus Reference Center (IHRC), more than 200 different HPV types have been described, each with a specific tissue tropism; only a small proportion of them have the potential to cause malignant transformation of human cells. Twelve of these HPV genotypes have been recognized by the International Agency for Research on Cancer (IARC) as causative for the development of cervical cancer, and eight others may play a role in the development of cervical cancer (12). HPV types are divided into high-risk (HR-HPV) and low-risk (LR-HPV) types according to their ability to cause more or less severe epithelial cell abnormalities. The most common HR-HPV types are HPV 16 and 18, which alone are responsible for around 70% of cervical cancer cases. In addition, other HR-HPV types play a role in some anogenital cancers (i.e., anus, vulva, vagina, and penis) as well as cancers of the head and neck, particularly the oropharynx in men and women. Among the LR-HPV types, HPV 6 and 11 are the most common and are responsible for 90% of cases of condyloma acuminatum (i.e., genital warts) (13, 14). Sexual behaviors such as oral and anal sex, a high number of sexual partners, same-sex intercourse and an age of sexual debut of 18 years or less are important risk factors for HPV-related cancers (15). According to GLOBOSCAN 2018 database, HPV is the second cause of infection-attributable cancer, with an estimate worldwide of 690,000 cases and age-standardized incidence rate of 8.0 cases per 100,000 person-years (16). Squamous cell carcinoma of the anus (SCCA) is the most common subtype of anal cancer, it is considered 100% associated with HPV infection and its rate is elevated in person living with HIV. An estimated 30,000 SCCA were diagnosed globally in 2020 (17). Oral HR-HPV infection ranks, along with smoking and alcohol consumption, among the most important risk factors for oropharyngeal cancer. Worldwide, 98,400 new oropharyngeal cancer cases were estimated in 2020, with an age-standardized incidence rate of 1.1 per 100,000; however, different values are found among regions (18). In addition, the incidence of vulvar cancer has increased in recent years, possibly due to increasing exposure to HPV (19); the GLOBOSCAN 2022 database reports a worldwide age-standardized incidence rate of 0.83 (20). Worldwide, cervical cancer (CC) is the fourth most commonly diagnosed cancer and the fourth leading cause of cancer-related death in women of all ages; the impact worsening in women aged 15–44 years, for whom cervical cancer ranks second in both diagnosis and cause of death (21). Globally, in 2020, 604,000 new cases and 342,000 deaths have been estimated, with respective age-standardized rates disproportionately elevated in High/Very High Human Development Index countries compared to countries with low/medium human development index (18.8 vs. 11.3 per 100,000 for incidence; 12.4 vs. 5.2 per 100,000 for mortality) (22). In Europe, CC rates are affected by internal variability: in fact, Eastern Europe compared to Western Europe has double age-standardized incidence rate (14.5 vs. 7.0) and triple aged-standardized mortality rate (6.1 vs. 2.0) (23). In women of all ages, CC is the ninth most diagnosed cancer and the tenth leading cause of cancer death; but in women aged 15–44, CC it is the third most common cancer, both in frequency of diagnosis and cause of cancer death (21). In Italy in 2022, 2.500 new diagnoses of CC are estimated, representing 1.3% of all cancers occurring in women. This percentage is as high as 4% in the youth group (women under 50 years old), where CC is the fifth most frequent cancer. In the same year, 2.500 total deaths are estimated for all uterine cancers (24).

## 3 Elimination of cervical cancer: a public health problem

In 2020, the World Health Organization (WHO) issued a document aimed at raising awareness about the elimination of cervical cancer, defining this neoplasia a preventable disease and its eradication a global health issue. HPV vaccination and cervical cancer screening are best-buy interventions, capable of contributing to the reduction of mortality rates, but they need to be improved to achieve the WHO’s goal (13, 25). The WHO’s global strategy calls on all countries to act to reduce the incidence of cervical cancer to less than four cases per 100,000 women per year by achieving 90% HPV vaccination coverage, 70% screening coverage, and 90% access to treatment for cervical precancer and cervical cancer, including access to palliative care. These 90–70-90 targets should be met by 2030 and, to achieve these goals, governments must invest in promoting communication, and improving the access to adequate information (26). Along these lines, in 2021 the European Commission underlined the need of developing new guidelines to implement the screening, diagnosis, treatment, rehabilitation and palliative care for cervical cancer in all member states (27) and a roadmap was then published to accelerate elimination of cervical cancer in Europe, giving technical assistance and promoting a common fight against cervical neoplasia (28). Focusing on Italy, the HPV vaccination coverage among eleven-year-old girls and boys, already significantly below the 95% target, further decreased in 2020, as well as the screening for cervical cancer, due to the COVID-19 pandemic impact. Henceforth arose the necessity to publish the new version of the national plan of vaccine prevention, which highlighted four main actions to be undertaken: (1) the national HPV vaccination campaign should be relaunched and strengthened; (2) access to vaccination services should be expanded; (3) determinants of vaccine hesitancy should be analyzed to intervene on theme; (4) integrated and coordinated disease management pathways should be developed to improve the quality of life of patients affected by neoplasia (29, 30).

### 3.1 Screening for HPV

**Figure 1:**
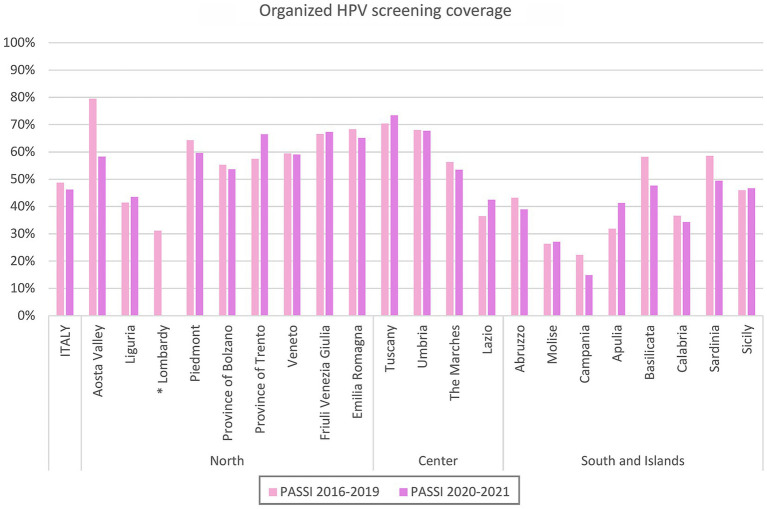
Organized HPV screening coverage, by region. Data from surveillance PASSI 2016–2019 and 2020–2021. ^*^2020–2021, missing data.

The basic and original idea of STI prevention in general is based on screening to identify and treat infected individuals immediately, before they develop complications and disease (i.e., secondary prevention). However, it is also important to identify, test, and treat sexual partners to prevent further transmission and/or reinfection. Screening for HPV-associated diseases can be done by cytology and/or HPV testing of cervical samples or cytology of anal samples (31). Concerning screening coverage, the majority of countries with screening programs reported reaching about 10 to 50% of the target population, with a significant concentration from the European and Western Pacific regions (32). Focusing on Europe, data are more reassuring, with several countries reporting screening rate exceeding 70% and between 50 and 70% in 2019. The percentage tends to decrease in Eastern European states, hence, more efforts are necessary to reach the WHO targets set for 2030 (33). Nowadays, in Italy, the Pap test (conventional cytology) and the papillomavirus test (HPV-DNA test) are both used for cervical cancer screening: the first is offered to younger women, between 25 and 30 year-old, every 3 years, the second to older women, between 31 and 64 year-old, every 5 years, as scientific evidence shows that nucleic acid tests are more cost-effective at this age (34). According to surveillance data collected by PASSI in Italy in the two-year period 2020–2021, 77.5% of women aged 25–64 were up to date with the cervical cancer screening program (i.e., Pap test or HPV-DNA test depending on age), but there were worrying differences between the north and south of the country. For example, HPV screening achieved the highest coverage in northern and central Italy (up to 90.1% in the autonomous province of Bolzano), whereas the Molise region achieved the lowest (62.3%) (35). In addition, a significant proportion of women interviewed in the same surveillance reported never having attended cervical cancer screening (12%) or having done so more than 3 years ago (12%); the most frequently cited reason for not attending screening was the misconception that it was not needed (35). When analyzing data on organized screening (i.e., excluding private screening), the heterogeneous pattern between Italian regions remained similar in both the 2016–2019 and 2020–2021 PASSI surveys (Figure 1).

### 3.2 Vaccination against HPV

**Figure 2:**
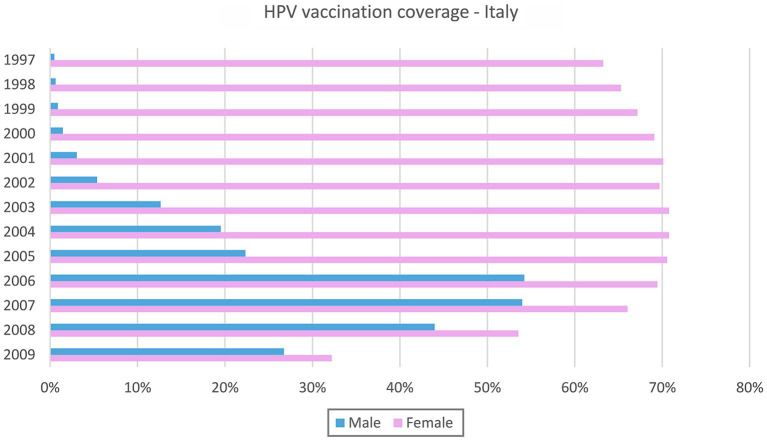
Italian vaccination coverage for the 1997–2009 birth cohorts, reported by sex.

The WHO recommends that HPV vaccination be included in all national public programs, as it is recognised to be effective in preventing cervical intraepithelial neoplasia (CIN), vulvar intraepithelial neoplasia (VIN), vaginal intraepithelial neoplasia (VaIN), and anal intraepithelial neoplasia (AIN) and their corresponding cancers (36). The global number of countries that has a national programme for HPV vaccination is still insufficient (36). Although HPV vaccine coverage is increasing, only 15% of girls received a complete cycle of vaccination worldwide (37). The majority of the low-and middle-income countries does not have a vaccination programme yet. This lack of primary prevention is relevant, considering that nine in ten cervical cancer deaths worldwide actually occurred in those low and middle income countries (36). In Europe, a completely distinct scenario prevails. Firstly, HPV vaccination is offered free of charge, which increases uptake depending on convenience (38) in comparison to other countries worldwide where HPV vaccination is only offered for a fee. In fact, although maternal willingness to pay for HPV vaccination increases with complacency about HPV-related diseases and their consequences, cost may have a negative impact on overall acceptance, especially among families of low socioeconomic status (39–41). Secondly, by 2019, most EU countries had already included HPV vaccination in their national immunization programs. Most current programs target prepubertal girls aged 9–14 years, either through organized school-based vaccination programs or though vaccinations administered by primary care providers (including general practitioners, nurses, and gynecologists). Since the decade 2010–2019, several countries have extended vaccination to boys of the same age, and more will follow (42). Despite the availability of a vaccine, the percentage of HPV-vaccinated girls still varies significantly across countries, ranging from 99% in Malta to 3% in Bulgaria, according to the latest updated data in 2021 (33). In Italy, HPV vaccination has been actively offered free of charge to women and men over the age of 12 since 2008 and 2015, respectively, (43). The available data on national and regional HPV vaccination coverage for the 1997 to 2009 birth cohorts (as of December 31, 2021) show a significant difference in vaccination coverage between boys and girls (Figure 2, Supplementary material) and between regions. In 2021, national HPV vaccination coverage rates for girls ranged from 32.2% in the youngest cohort (2009) to 70.8% in the 2003 and 2004 cohorts, while for boys, national coverage rates ranged from 0.5% in the older cohort (1997) to 54.2% in the 2006 cohort. Nevertheless, the disruptive impact of the pandemic on health services and thus on normal vaccination adherence, is quite evident and has yet to be fully addressed (44, 45). Despite the improvements achieved in the individual cohorts, the average HPV vaccination coverage rate in girls and boys is still far from the expected optimal threshold envisaged by the Italian national vaccination prevention plan (i.e., 95% at age 12), and there is an obvious problem of inequality between regions as recently highlighted (45, 46).

## 4 Discussion

In Italy, to address the challenges of health promotion and HPV prevention and to ensure the achievement of equitable interventions, priority actions such as the development of health policies in collaboration with a coordinated and integrated network of professionals and stakeholders, are needed as highlighted in the 2020–2025 Italian National Prevention Plan. Efforts should be made in order to mitigate interregional disparities, to promote education and information at every level, from schools to healthcare professionals. In addition, in the 2023–2025 National Vaccination Prevention Plan, recently adopted by the Conference of Government and Regions (29), the Italian Ministry of Health has renewed the goal of strengthening the three-arm prevention of cervical cancer and other HPV-related diseases, with the aim of providing the same level of primary care (LEA – Livelli Essenziali di Assistenza) (47). Mobilization, health education, and counseling are crucial to ensure high vaccination coverage, extensive screening coverage, and strong adherence to treatment (48). The role of healthcare providers is essential in refuting the myth on HPV and cervical cancer and in ensuring adequate dissemination of information, starting from defining cervical neoplasia as a preventable disease, to conveying to families and individuals the importance of prevention through a safe and effective vaccine and through the identification of preneoplastic lesions (48). Improving the ability of providers to communicate about these themes may help in reducing vaccine hesitancy (49) which is still deeply entrenched within the population and associated with lack of information about HPV and its effects, as well as HPV vaccination (50). Another fundamental strategy to implement is certainly reproductive health and sexual education of adolescents (51), that unfortunately in Italy still remains critical and regionally inhomogeneous (52), and much lower than in other European countries (53). According to the Italian National Prevention Plan, the school is a crucial place for health promotion as it can ensure a continuous educational approach throughout the school years and enable strong and effective interaction with local health services. Several studies have shown that sexual education of adolescents has a positive impact not only on the use of contraceptive and thus on the reduction of STIs and unwanted pregnancies, but also on the prevention of violence, the development of healthy relationships and the strengthening of social and emotional awareness (54). In Italy sexuality education is not a compulsory subject in either primary or secondary school curricula, so there are many different providers with different methods and objectives (52). Although the high prevalence of sexually transmitted infections among adolescents is partly due to a combination of biological, behavioral and cultural characteristics typical of this age (55), the social network of adolescents and their referral figures (51, 54) as well as potential barriers to accessing health services (56), age of first sexual contact (55) and increasing life expectancy must also be considered, all of which contribute to an increased risk of acquiring sexually transmitted infections. Although there is clear evidence that vaccination is the most effective long-term intervention in both sexes and that cervical cancer screening is an essential component of secondary prevention, we still need to strengthen and improve sexuality education policies at national level to fully achieve the adequate standards required by the 2030 Agenda and reduce inequalities not only between Italian regions but also between Italy and other European countries.

## Data availability statement

Publicly available datasets were analyzed in this study. This data can be found at: https://www.epicentro.iss.it/passi/dati/screeningcervicale, https://www.salute.gov.it/portale/documentazione/p6_2_8_3_1.jsp?lingua=italiano&id=27.

## Author contributions

SG: Data curation, Formal analysis, Writing – original draft, Conceptualization, Visualization. FV: Data curation, Writing – original draft, Conceptualization, Supervision. AS: Writing – original draft, Conceptualization. LD: Supervision, Writing – review & editing. LB: Data curation, Supervision, Writing – original draft, Writing – review & editing, Conceptualization.
