# Supplementary material for: Sexually transmitted infections and the HPV-related burden: evolution of Italian epidemiology and policy
Source: Front Public Health. 2024 Mar 15;12:1336250. doi: 10.3389/fpubh.2024.1336250 (PMC10978588; doi:10.3389/fpubh.2024.1336250)
Supplement: Supplementary file 1 [file Data_Sheet_1.PDF]

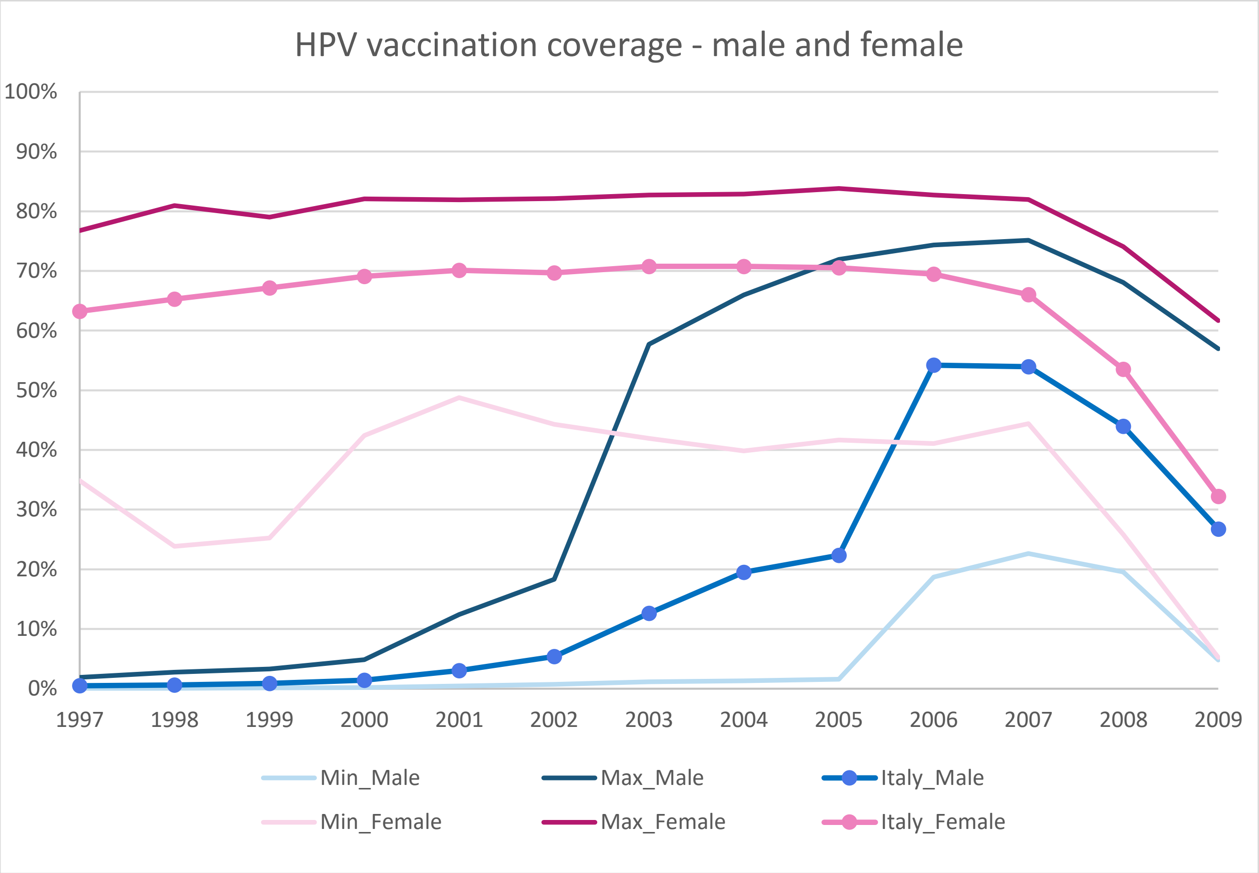

Figure 3. Italian male and female vaccination coverage for the 1997-2009 birth cohorts, reported as minimum and maximum level of coverage for each birth cohort and by sex at national level.
